# Emerging and Re-Emerging Leishmaniases in the Mediterranean Area: What Can Be Learned from a Retrospective Review Analysis of the Situation in Morocco during 1990 to 2010?

**DOI:** 10.3390/microorganisms8101511

**Published:** 2020-09-30

**Authors:** Kahime Kholoud, Lahouari Bounoua, Denis Sereno, MoulayAbdelomain El Hidan, Mohamed Messouli

**Affiliations:** 1 Laboratory of Applied Sciences for the Environment and Sustainable Development, School of Technology Cadi Ayyad University, 40000 Marrakesh, Morocco; 2 Research Group on Impact, Vulnerability and Adaptation to Climate Change in Morocco (GRIVAC), LHEA, Faculty of Sciences Semlalia, Cadi Ayyad University, 40000 Marrakesh, Morocco; 3 Biospheric Sciences Laboratory, NASA’s Goddard Space Flight Center, Maryland, MD 21041, USA; lahouari.bounoua@nasa.gov; 4 IRD, University of Montpellier, InterTryp, 34000 Montpellier, France; 5 Science and Technique Center, Ait Melloul, Ibn Zohr University, 800000 Agadir, Morocco; elhidan@gmail.com; 6 Research Group on Impact, Vulnerability and Adaptation to Climate Change in Morocco (GRIVAC), LHEA, Faculty of Sciences Semlalia, Cadi Ayyad University, 40000 Marrakesh, Morocco; messouli@gmail.com

**Keywords:** leishmaniases, cutaneous leishmaniases, visceral leishmaniases, *Leishmania major*, *Leishmania tropica*, *Leishmania infantum*, epidemiology, Morocco, Sandfly

## Abstract

In Morocco, cutaneous and visceral leishmaniases represent a public health concern. In this opinion paper, we propose to highlight chosen elements that have governed the drastic increase in the incidence of leishmaniases recorded in Morocco during the period between 1990 to 2010 in order to guide the prediction of the expansion of diseases and epidemic events. We highlight that the dispersion of the zoonotic cutaneous leishmaniasis (ZCL) form, caused by the *Leishmania major* parasite, appears to be closely related to that of its arthropod vector density, which is sensitive to changes in climate. The dissemination of anthroponotic cutaneous leishmaniasis (ACL) was related to an increase in human travel and local tourism during the studied decades. These are linked to economic expansion and infrastructure development. Interestingly, the main ACL foci are spatially aligned with the highways, and their occurrence was synchronized with the building of transportation infrastructure. During the above-mentioned decades, the zoonotic visceral leishmaniasis (ZVL) caused by *Leishmania infantum* has expanded from its historical northern territories, dispersing outwards in all directions. This spread follows the emergence of hamlets and villages connecting with major cities.

## 1. Introduction

**Figure 1:**
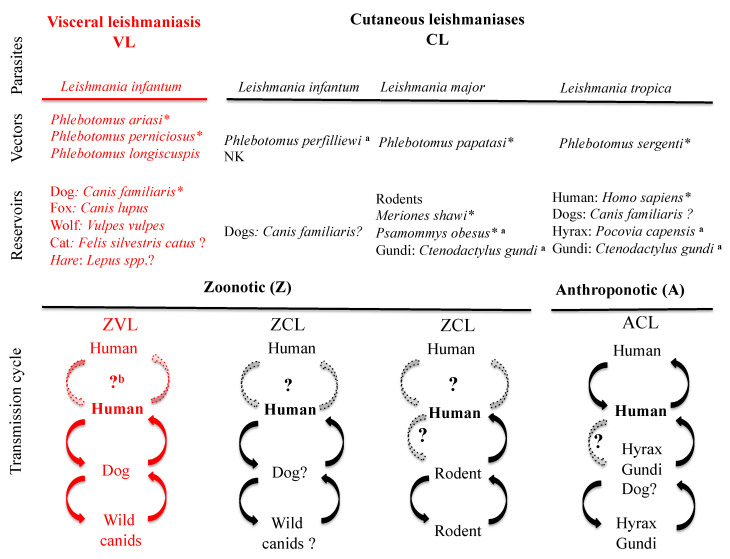
Schematic representation of the various transmission cycles of Leishmania species with a medical impact in Morocco. NK: not known. Dashed lines represent unconfirmed/unknown transmission pathways. ? indicates uncertainty. * indicates proven vectors or proven reservoirs, according to the rules that define them. a: not currently demonstrated in Morocco. b: occurs in human immunodeficiency virus patients (HIV+) and in drug users [16].

Leishmaniases are a worldwide health problem and a scourge for those with limited means to combat them. These diseases rank only after malaria in terms of annual incidence, with a mortality between 20,000 to 40,000 persons annually worldwide [1], and are a public health concern in Morocco [2]. Currently, the use of chemotherapy to treat these infections is challenged by the limited number of available molecules and by the emergence of chemoresistant parasites [3,4,5,6,7]. Epidemiological cycles of leishmaniases, as illustrated in Figure 1, are subdivided into two broad categories: the zoonotic forms of leishmaniases (ZL), where the primary reservoirs are wild or domestic mammals, and anthroponotic forms (AL), for which humans are the primary reservoirs. Two main clinical forms can be distinguished: visceral (VL) and cutaneous (CL) [8]. In Morocco, zoonotic visceral leishmaniasis (ZVL) is caused by *Leishmania infantum* with dogs as the primary reservoir, and three *Phlebotomus* species—*P. ariasi*, *P. perniciosus* and *P. longicuspis*—as vectors [9,10]. Cutaneous leishmaniases are caused by *Leishmania major*, *Leishmania tropica* and *L. infantum*. Zoonotic cutaneous leishmaniasis (ZCL)—caused by *L. major*—involves small rodents as a reservoir and *P. papatasi* as a vector [11]. In addition, ZCL is sporadically caused by *L. infantum,* with a less well-known epidemiological cycle [12,13]. Anthroponotic cutaneous leishmaniasis (ACL) is caused by *L. tropica* and is transmitted by *P. sergenti* with humans as reservoirs [14]; nevertheless, a zoonotic cycle may eventually be present [15].

## 2. Incidence of Leishmaniases in Morocco; Review of the Period 1990–2010

**Figure 2:**
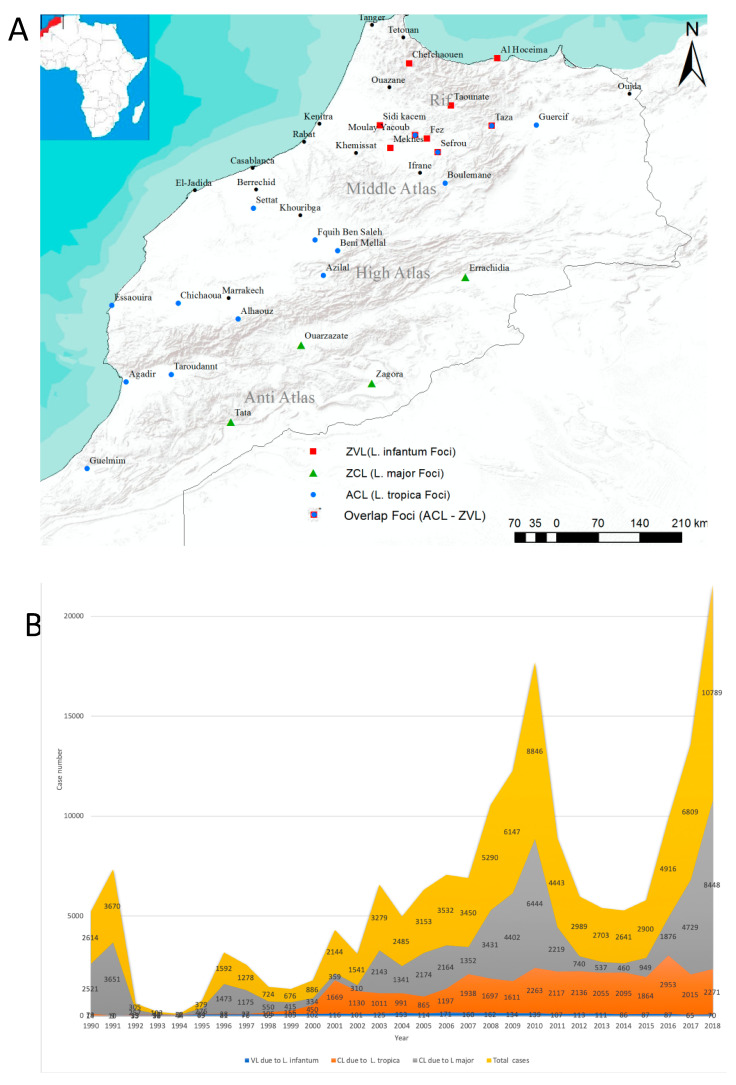

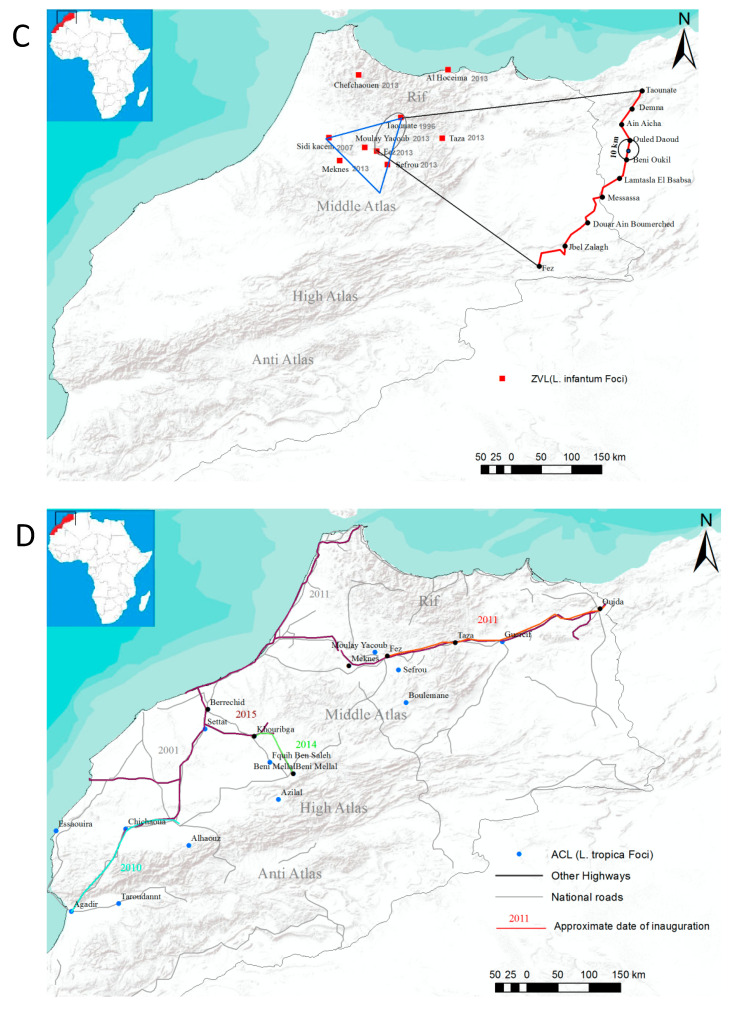
Incidence and dispersal of leishmaniases in Morocco. Schematic representation of the distribution of leishmaniasis in Morocco, according to the clinical forms (**A**). Total leishmaniases (zoonotic cutaneous leishmaniasis (ZCL), anthroponotic cutaneous leishmaniasis (ACL) and visceral leishmaniasis (VL)) in Morocco from 2000 to 2018. Case number sand total cases are given on the graph (**B**). Distribution of ZVL (*L. infantum*) in relation with the dispersal potential of reservoir hosts (**C**). Distribution of ACL (*L. tropica*) in relation with the development of the road infrastructure (**D**). NB: ZCL caused by *L. infantum* is not represented in the graphs.

Morocco is localized in northwestern Africa (Figure 2A) and is characterized by a Mediterranean climate with hot and dry summers and temperate to mild winters in the coastal areas. The country’s interior is cool to cold on the Atlas mountains, the Rif and the highlands of the eastern regions [17]. In southern Morocco, south of the anti-Atlas, the coastal areas are under oceanic influence, while away from the coast, the climate is arid [17]. In 2015, the total number of leishmaniases cases was 949 of ZCL, 1863 of ACL and 86 of VL [18]; in the decade from 2000 to 2010, leishmaniases have had an alarming impact, as illustrated by the data gathered from the official records from bulletins and reports of the National Directorate of Epidemiology and Disease Control of the Ministry of Health [19] and by data gathered from the “Performances du Programme National de Lutte Contre les Maladies Parasitaires”. From 2001 to 2010, about 38,500 CL cases caused by *L. major* and *L. tropica* and about 1300 VL cases caused by *L. infantum* were declared. The temporal trend in 1991–2000 and 2001–2010 showed a three-fold increase in the number of ZCL infections. Anthroponotic cutaneous leishmaniasis increased by about 17-fold and ZVL increased by about two-fold over the same periods (Figure 2B). The total number of CL cases due to *L major* underwent a sharp decrease between 1990 and 1994 and, except for 1996 and 1999, it remained below 1000 cases per year until 2002. After 2002, it increased steeply until 2010, reaching 6444 cases. Cutaneous leishmaniasis due to *L. tropica* and VL due to *L. infantum* have both increased monotonically during the same period. Starting from 2000 cases, infections caused by *L. tropica* and *L. infantum* increased slowly at a rate of 1.15% and 0.48%, respectively, while cases caused by *L major* experienced an exponential increase (Supplementary Data S1). From 2010 to 2018, after a sharp decrease in ZCL cases, the situation began to be dramatic. For ACL and ZVL, the monotonical increase was maintained (Figure 2B). After a sharp decline in ZCL case numbers after 2010, a new epidemic situation has arisen (see Figure 2B).

## 3. What Can We Learn from the Past Moroccan Situation?

If, during the early 1990s, the geographic location of each individual clinical form (VL and CL) was defined by its nosogeographical entity and appeared to be confined to its historical foci [20], after 1999, a wide spatial dispersion was observed across the country.

### 3.1. Zoonotic Cutaneous Leishmaniasis due to L. major

Historically, ZCL due to *L. major* has been restricted to the Errachidia province in south-eastern Morocco. A high incidence is still recorded in the Saharan desert and the arid steppes that fringe the northern border and the Middle East [21,22]. In these sub-Saharan regions, *Phlebotomus papatasi* (proven primary vector) and *Meriones shawi* (primary reservoir) find adequate ecological conditions. The palm groves provide food and shelter for reservoirs and insect vectors of *L. major*. The increase in rodent activity and their migration are responsible for the increase of ZCL cases and the northern extension of the disease [22,23]. While the reservoir hosts thrive in relatively cooler regions, *P. papatasi* cannot survive outside the temperature range of 10 °C to 40 °C [24], and its reproduction is hampered at temperatures below 15 °C [25,26], limiting the propagation of ZCL (Figure 2A). On the south of the high Atlas Mountains, the recorded annual mean temperature and humidity profiles fit the physiological characteristics of *P. papatasi* well. There, ZCL foci were evidenced in the 1980s [27] in Zagora and Tata (Figure 2A), whereas in the north and the northeast, in Ouarzazate and Errachidia, important foci appeared in the 2000s [28]. The oases of Ouarzazate and Errachidia experienced a slight increase in the mean annual minimum temperature in the decades 1990–1999 and 2000–2010, which was associated with an increase in the incidence of all major foci. The link between the minimum surface temperature and the incidence of ZCL due to *L. major* has been documented [26] and should also have governed the increase in ZCL incidence in the Ouarzazate area during the 1990s and 2000s. The Atlas extends from the Atlantic Ocean, south of Agadir, to the northeast and includes the anti-Atlas and the high Atlas. With an elevation mean of 3050 m, the average temperature recorded is below 0 °C, making it an obstacle to the northward propagation of the vectors and the reservoirs of *L. major*. Accordingly, *P. papatasi* is highly abundant between 400 m and 600 m of altitude [29] but has occasionally been trapped at up to 1800 m of elevation [30]. Minimal temperature and humidity also affect incubation (the time required for the development of the infectious agent in the body of the vector) and maturation periods, thereby affecting the vectorial capacity of *P. papatasi* populations [25]. Other underlying factors might also impact the capacity of *P. papatasi* populations to transmit *L. major* in Morocco, restricting ZCL to the southern part of the Atlas Mountains [31]. Altogether, seasonal climate prediction would help to track the location and timing of ZCL outbreaks in Morocco [26].

### 3.2. ZCL due to L. infantum

The incidence of zoonotic cutaneous leishmaniasis caused by *L. infantum* is limited in Morocco [32], and its cycle of transmission is not entirely known (Figure 1); therefore, the analysis of factors which play a role in the dynamic of the disease is not possible.

### 3.3. ACL due to L. tropica

Anthroponotic cutaneous leishmaniasis due to *L. tropica*, also known as the urban form of leishmaniasis, was originally widespread in the semiarid provinces of the central and western slopes of the Atlas, from the regions of Azilal in the center up to Essaouira in the west, and Agadir-Guelmim in the south [33]. Currently, ACL has extended in all directions and overlaps with some ZVL foci [34,35,36].

Since *P. sergenti*, the primary vector of *L. tropica*, is present in all Moroccoan ecoregions, one can speculate that the spread of ACL to new foci is likely to be linked to human travel [37]. Therefore, it has the potential to have a large geographical dispersion. In Morocco, before 1999, outbreaks were recorded in rural urban and suburban areas, such as Taza and Guercif for the northern regions, Azilal in the middle and Essaouira-Agadir in the south (Figure 2C). The spread of ACL between rural and large urban areas—Fez–Moulay-Yacoub, for example—might have occurred via the development of larger and more affordable mass ground transportation infrastructure (roads or trains). The economic development recorded during this period led to an increase in human displacement in search of commerce and other business and leisure activities. Interestingly, the inauguration of new national roads and highways—Meknes–Fez–Taza–Oujda in 2011, Casablanca–Marrakech–Agadir (2001–2010), Khouribga–Beni-Mellal (2014) and Berrechid–Khouribga (2015)—has linked all major ACL foci (Figure 2C). These roads have facilitated the movement of populations, including infected ones. A new ACL focus has emerged in Settat in 2015 following its connection by highway to Casablanca–Marrakech–Agadir. The first ACL focus in Morocco was reported in Azilal in 1989 [38] concomitantly to the expansion of the transportation pathways; foci were declared in Essaouira, Agadir and Guelmim in 1991 [39] and in Taza and Guercif in the northeast in 1997 [40]. Following the additional expansion of the road network in 2000, other new major foci have emerged in Sefrou and Moulay-Yacoub to the northeast, in Beni-Mellal and Fquih-Ben-Salah in the center and Chichaoua and El-Haouz. Further risk of ACL foci development in El-Jadida and Casablanca is present following their connection to the old foci of Marrakech and Agadir in 2010.

### 3.4. ZVL due to L. infantum

In Morocco, ZVL was initially confined to the northern province of Taounate but has since spread to other territories [10,41,42]. *P. ariasi*, *P. perniciosus* and *P. longicuspis* are vectors of *L. infantum* [40,43]. *P. ariasi* and *P. perniciosus* have preference for sub-humid to humid bioclimates. *P. ariasi* is present in altitudes ranging between 1000 m and 1400 m, and *P. perniciosus* has a high density at altitudes below 1000 m. *P. longicuspis* inhabits dry areas between 800 m and 1000 m of altitude [30,33,41]. In the northwestern part of Morocco, the average minimum temperatures of below 10 °C from November to April limits the emergence and abundance of sandflies, lowering the risk of outbreaks. Sandflies survive well near human dwellings and livestock shelters, where they feed on domestic dogs (*Canis familiaris*), a reservoir of *L. infantum* [13,44,45]. In Morocco, the first documented case of canine leishmaniasis was published in 1932 [46]. Beside domestic dogs, stray dogs are also reservoirs for *L. infantum* [40,45,47]. The ecology of stray dogs and home range areas provides information on the dispersal potential of these *L. infantum* reservoirs. Most stray dogs are engaged in random free-ranging behavior, such as looking for food. Dogs are social animals, and in their quest for food, urban and suburban areas provide a favorable environment. Except for being killed by cars or humans, stray dogs have no predators in these suburban territories. Because of their rapid and short reproductive cycle, with an average of five to six puppies, the population number of stray dogs generally grows faster than its food supply. Unlike other wild animals, which defend their territories, when this condition arises, stray dogs disperse freely towards other suburbs [48,49]. The home range of stray dogs is about 4 to 200 km^2^ [50], delimiting a dispersion circle with a maximum radius of 10 km. This range does not conform to rigid boundaries but varies with location (urban versus open fields) and food availability [49].

In Morocco, the first VL foci were documented in Taounate in 1988 [32]. Subsequently, new foci were observed in the province of Sidi-Kacem in 2007 [28], and in the urban conglomerations of Taounate in 2013 (Chefchaoun, Taza, Fez, Moulay-Yacoub, Meknes, Sefrou and Al-Houceima) [51]. The distance between these urban centers and Taounate is about 100 km. They are connected with each other by small towns and villages (Figure 2D). As a first order of approximation, and considering that ZVL has spread from Taounate outward to these surrounding urban centers, this would have been done through stray dog dispersion circles. Important ZVL foci appear to be within a triangle limited by the urban areas of Taounate, Sefrou and Sidi-Kacem, with Fez in the center. This area is surrounded by large cities, such as Meknes and Taza, and by villages. Between Fez and Taounate, several villages that are distant from each other by 7–13 km are present (Jbel-Zalagh, Douar-Ain-Boumerched, Messassa, Lamtasla-El-Bsabsa, Beni-Oukil, Ouled-Daoud, Ouled-Said, Ain-Aicha, Demna), along the N8 national road (Figure 2D). Other hamlets of smaller size connect these towns and villages, further reducing the distances between suburban settlements and increasing their connectivity. We argue that, via these connectivity circles, ZVL might have propagated from Taounate to the foci of Meknes and Sidi-Kacem via Fez, with stray dogs carrying the infection across hamlets and villages in their quest for food and shelter. The city of Taza (at 600 m of altitude) is not directly connected to Taounate by a main road but is connected to Fez via the N6 and since 2011 by the expressway A2 (Highway of Morocco website, in French; http://www.adm.co.ma). Taza’s elevated location in the Rif and the low level of connectivity of towns between Taounate and Taza has probably limited canid dispersion from Taounate, supporting the observation of the absence of ZVL foci between these two urban centers. In addition, the minimum temperatures in Taza remained below 15 °C between mid-June to mid-October, severely limiting the vector’s reproduction capability [25]. Finally, the low connectivity over long distances and rough topography between Taounate andAl Hoceima and Taounate and Chefchaouen means that these axes are not preferential for ZVL propagation (Figure 2D).

Between 2003 and 2013, 20 cases of ZVL were recorded in Tetouan, with one in Tanger and 12 in Ouazane, all located north of Taounate (Figure 2B); five cases were recorded in Guercif and one in Oujda in the east, while in Khemissat—233 km west of Fez—14 cases were recorded. In Ifrane, south of Fez, one case was recorded. In Beni-Mellal—450 km away from Taounate—33 cases were recorded. All of these provinces were ZVL-free prior to 1990 and have not yet been declared as ZVL foci.

## 4. Conclusions

Zoonotic cutaneous Leishmaniasis is caused by *L. major*. Although *P. papatasi* has a wide geographic dispersion in Morocco, the physiological requirement to thrive means that high densities are observed in geographically limited areas, mostly in the south of the high Atlas, where *Meriones shawi* thrives. Subtle changes in surface climatic conditions would affect the vector density/activity and therefore impact potential outbreaks. Studies suggest that changes in surface climatic conditions may have already initiated a trophic cascade that has resulted in an increasing incidence of ZCL caused by *L. major*. The argument is that an increase in precipitation during the last decade has increased local vegetation, providing more food and shelters for both the rodents, a reservoir of *L. major*, and sandflies that transmit it. Interestingly, the increase in minimum temperatures that has allowed more sand fly larvae to survive through the winters might have created endemic ZCL conditions that did not previously exist in the pre-Saharan regions of Morocco.

Anthroponotic cutaneous leishmaniasis is caused by *L. tropica*. The ecological plasticity allows *P. sergenti* to adapt to diverse bioclimatic zones and ecoregions, from arid to semi-arid areas of Morocco. The primary reservoir for *L. tropica* is humans. Population growth and the economic expansion of Morocco have drastically stimulated human travel and local tourism across Morocco, thereby favoring the dispersion of ACL. Both in the northern and southern parts of the country, major ACL foci are aligned with roadways, and the chronology of their occurrence appears to be in line with the construction of the transportation infrastructure.

Zoonotic visceral leishmaniasis is caused by *L. infantum*. In Morocco, visceral leishmaniasis has expanded from its historical northern territories and propagated outwards in all directions. Indirectly related to increases in population, ZVL appears to propagate with the emergence of small towns, villages and hamlets around major urban centers, where its main reservoir—stray dogs—proliferates. We argue that the southward propagation of ZVL from its original northern location of Taounate follows the development of small hamlets and towns connecting it to other major cities. These connections appear to create dispersion circles and free-range metrics that fall within those reported in the general literature of stray dogs’ ecology.

## Supplementary Materials

Supplementary materials can be found at https://www.mdpi.com/2076-2607/8/10/1511/s1.
