# Supplementary figures and images for: Emerging and Re-Emerging Leishmaniases in the Mediterranean Area: What Can Be Learned from a Retrospective Review Analysis of the Situation in Morocco during 1990 to 2010?
Source: Microorganisms. 2020 Sep 30;8(10):1511. doi: 10.3390/microorganisms8101511 (PMC7650785; doi:10.3390/microorganisms8101511)

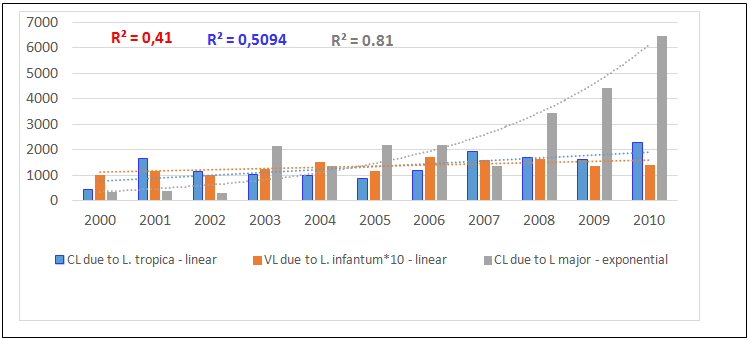

Supplement: Supplementary file 1 [file microorganisms-08-01511-s001.zip › SuppdataS1.tif]
